# Coding and Non-coding RNAs, the Frontier Has Never Been So Blurred

**DOI:** 10.3389/fgene.2018.00140

**Published:** 2018-04-18

**Authors:** Florent Hubé, Claire Francastel

**Affiliations:** ^1^Université Paris Diderot, Sorbonne Paris Cité, Paris, France; ^2^Epigénétique et Destin Cellulaire, Centre National de la Recherche Scientifique UMR7216, Paris, France

**Keywords:** messenger RNA, non-coding RNA, bifunctional RNA, transcript isoform, UCSC genes, Affymetrix U133 Plus2 array, NCI-60 panel

In molecular biology, one of the founding dogmas states that genetic information, stored in the form of DNA molecules in the majority of living organisms, is translated into proteins via transient intermediaries, the RNAs. However, over the past 50 years, an increasing number of studies have highlighted that RNAs have a more significant and broader role. Whereas all the genomes of living organisms, whether single-cell or multi-cellular, have a high proportion of loci that are transcribed into RNA, this transcript is not necessarily translated into protein but can perform functions within the cell as the form of RNA. As a consequence, RNAs translated into proteins were named messenger RNAs (mRNA) as opposed to those that do not, which are referred to as non-coding or regulatory RNAs (ncRNA), clearly discriminating RNAs according to their protein coding capacity. Until recently, the coding/non-coding distinction appeared to be obvious, although it became more blurred in recent years. The first hints emerged when it became evident that the repertoire of genome-encoded RNAs is far more extensive and complex than previously thought. Indeed, many RNAs, here referred to as bifunctional RNAs (bifRNA), have both regulatory/non-coding and coding functions. The balance between non-coding and coding RNAs levels is modulated depending on the stage of development or differentiation, environmental cues, disturbances caused by a pathogen, etc. After having re-defined this striking but expanding class of bifRNAs, we will propose an estimate of the fraction of the human transcriptome that they may represent.

## The concept of bifunctional RNAs

**Figure 1:**
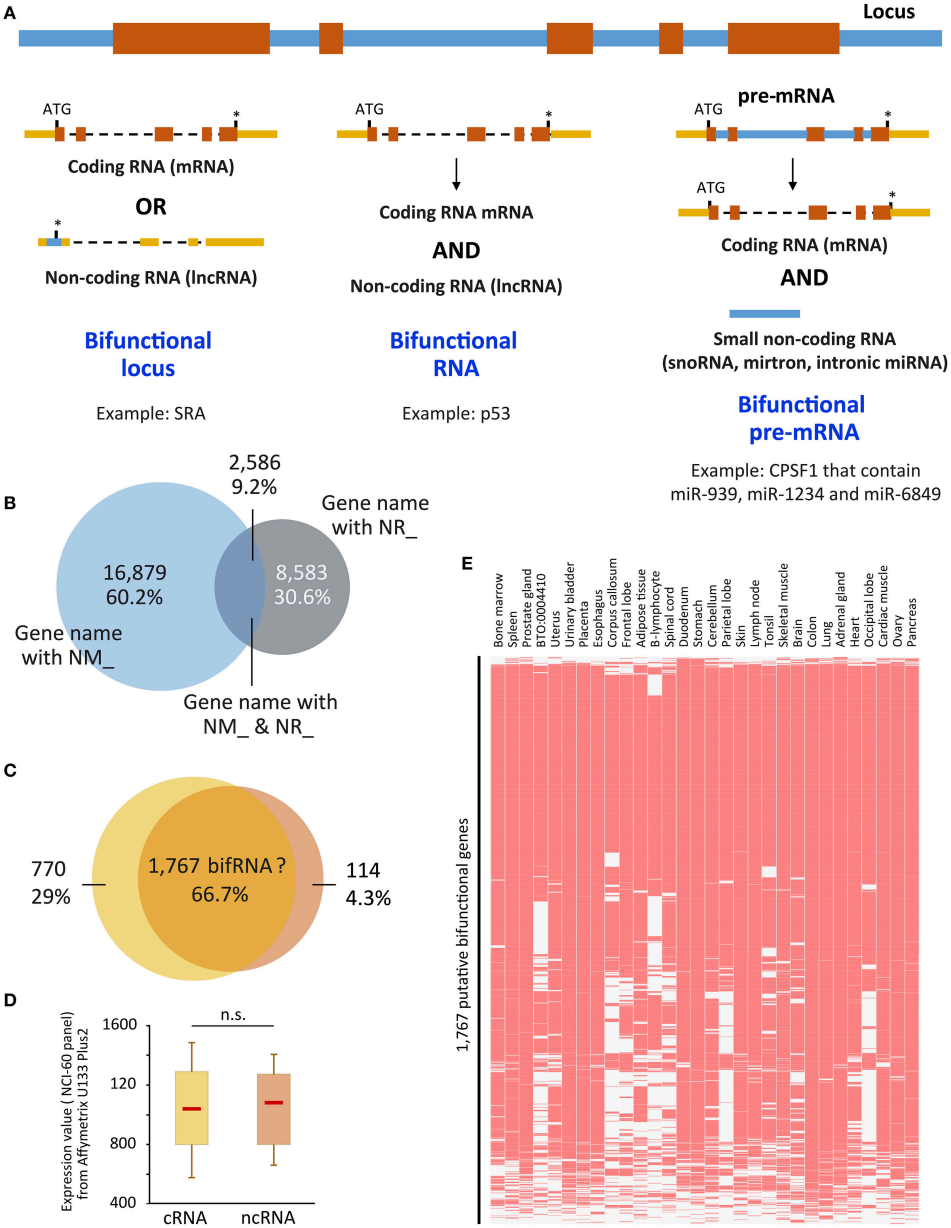
**(A)** Representation of the three ways to achieve bifunctionality. Genomic/intronic regions are in blue, exonic regions are in orange (coding sequences) or yellow (non-coding sequences). **(B)** Overlap between genes that present NM_ and NR_ isoforms. “UCSC genes” track from UCSC Genome browser was retrieved and NM_ and NR_ transcripts were sorted using Galaxy tools. Venn diagram was performed using the gene names. 16,879 (60.2%) and 8,583 (30.6%) were related to only NM_ (coding) or NR_ (non-coding) transcripts, respectively, whereas 2,586 (9.2%) hold both coding and non-coding isoforms. **(C)** Putative bifunctional RNAs are recognized by probes from Affymetrix U133 Plus2 array. The 2,586 genes were recognized by 5,635 probes, of which 66.7% were not able to distinguish between coding and non-coding isoforms (NM_ and NR_, respectively). The 66.7% correspond to 1,767 genes. **(D)** Expression values attributed to each probe corresponding to the 770 and 114 exclusively coding and non-coding genes, was retrieved from the NCI-60 panel that referred to a panel of 60 Human Tumor Cell Lines Screen related to nine different cancer types [Geo DataSet Record GDS4296, (Barrett et al., 2005)] and plotted as boxplots. Significance was assessed using Wilcoxon-Mann-Whitney test. **(E)** The expression of the 1,767 genes which coding and non-coding properties are not distinguishable was assessed in the Jensen Tissues (https://tissues.jensenlab.org/) using Enrichr website (http://amp.pharm.mssm.edu/Enrichr/). ATG, start codon; ^*^, stop codon; n.s., not significant; snoRNA, small nucleolar RNA, miRNA, microRNA; p53, protein 53 (Candeias et al., 2008); CPSF1, Cleavage and polyadenylation specificity factor 1 (Hube et al., 2017); cRNA, coding RNA; ncRNA, non-coding RNA. NM_ and NR_ are the prefixes used by RefSeq to describe protein-coding and non-protein-coding transcripts, respectively.

When we refer to bifRNAs, we assume that the same molecule is able to perform both functions, namely encode for a protein and possess its own regulatory function, but this term is often misused (Figure 1A). In fact, this is the case for a subset of them, such as SRA (Steroid Receptor RNA Activator), the pioneer member of this family [for review see (Ulveling et al., 2011b)]. Other examples have been described; SgrS (SuGar transport-Related sRNA) RNA partially inhibits glucose transporters mRNAs through base-pairing, and encodes a small polypeptide that prevents glucose transport (Maki et al., 2010; Vanderpool et al., 2011; Wadler and Vanderpool, 2007). The interaction between E3 ubiquitin ligase Mdm2 and p53 proteins usually drives p53 for degradation by the proteasome. Upon stress, the p53 mRNA was shown to also interact with the Mdm2 protein to both promote p53 synthesis and prevent Mdm2 from targeting p53 degradation (Candeias et al., 2008; Naski et al., 2009). A competition between translation and the structural function of RNA has therefore to be envisioned, although the way this competition is controlled remains rather obscure to date. However, one can imagine that sub-cellular localization is one important aspect to take into account, the translation being accomplished only in the cytoplasm whereas many ncRNAs are sequestered in the nucleus. Other bifRNAs have the extraordinary ability to separate the two functions in space and time, as it is the case for Oskar. The site of Oskar RNA and protein localization within the oocyte determines where germ cells form in the primordial embryo and where the abdomen develops (Rongo et al., 1995). This localization is controlled by a feedback loop in which Oskar mRNA is locally translated into a protein, which in turn maintains the localization of its mRNA. Strikingly, it was also shown that Oskar could act as a non-coding RNA at earlier stages in oogenesis, independently of its coding capacity, where it may play a structural role for the assembly of cytoplasmic complexes essential for development of the oocyte (Jenny et al., 2006).

In fact, for many bifRNAs that were described here or elsewhere (Hube et al., 2006, 2011; Xu et al., 2008; Hashimoto et al., 2009; Ulveling et al., 2011a,b; Nagano et al., 2015; Nam et al., 2016; Williamson et al., 2017), it is not the same RNA molecule that carries both coding and non-coding functions. The use of alternative splicing or alternative promoter account for the numerous mechanisms that allow the production of coding or non-coding isoforms from the same loci. In this particular case, the terminology of bifRNA is incorrect and misleading; the genomic locus is bifunctional, not the RNA molecules produced.

A third possibility must be addressed to conclude this discussion about the concept of bifunctionality. For example, in the case of all the small nucleolar RNAs (snoRNAs) that have been described so far as being exclusively of intron origin, at least in humans, the host precursor RNA belongs to the bifunctional class as it can produce, after splicing, a mature mRNA and a mature snoRNA, with distinct functions; snoRNAs guide chemical modifications to other RNAs whereas mature mRNAs can then be translated into a protein product.

## A fuzzy frontier between mRNAs and ncRNAs

The first non-coding RNA ever identified was the alanine (Ala) transfer RNA (tRNA), purified from yeast, whose structure was published in 1965 (Holley et al., 1965). This Ala-tRNA, like the other tRNAs, is involved in protein synthesis by carrying the amino acid Ala to the protein chain being synthesized. Since this discovery, thanks to advances in sequencing techniques, thousands of non-coding RNAs have been identified in the genomes of prokaryotes and thousands in that of eukaryotes.

In prokaryotic organisms, the majority of the genome is coding. In eukaryotes, the opposite is true: most of the genome does not encode proteins, but contains colossal information in the form of panoply of regions transcribed into functional RNAs of different sizes and functions. Only about 2–5% of mammalian genomes contain information to produce proteins, whereas about 90% is transcribed over the lifespan into a large and complex transcriptome of ncRNAs. The repertoire of these transcripts is the subject of an active international search. It is still incomplete, but already makes it possible to propose a novel picture of the RNA world.

By nature, an mRNA is defined by the coding sequence it contains. Typically, upstream and downstream of their coding sequences, mRNAs also contain transcribed but untranslated regions (5′- and 3′-UnTranslated Region, 5′- and 3′-UTR, respectively) that are highly structured. Both UTRs are known to play key roles in post-transcriptional regulation, including the control of mRNAs transport, the translation efficiency, the subcellular localization and the overall stability of transcripts (van der Velden and Thomas, 1999; Bashirullah et al., 2001; Jansen, 2001) What needs to be emphasized here is that, even mRNAs defined to code for proteins have secondary structures or sub-structures that are functional in the RNA form. Conversely, regulatory RNAs with functional properties linked to their sequence and folding can also carry sequences that are translated into peptides under specific physiological conditions (Kondo et al., 2010; Kageyama et al., 2011; Magny et al., 2013; Zanet et al., 2015).

Thus, many mRNAs may act as regulatory RNAs whereas more and more regulatory RNAs, first classified as non-coding, are shown to hide small coding sequences *i.e*. less than the 300nt/100aa limit defined so far (Dinger et al., 2008; Ulveling et al., 2011b). A strict discrimination between these two classes of molecules appears even less realistic since, in eukaryotes, some ncRNAs have features comparable to that of mRNAs, such as polymerase II-dependant transcription and addition of a cap and a polyadenylated tail for instance (Dinger et al., 2008; Kondo et al., 2010; Ulveling et al., 2011b), and even their presence in polysome fractions (Ingolia et al., 2011). In various mammals, from mice to humans, many of these long ncRNAs contain coding sequences that may be expressed in specific contexts. How, in that case, could they be distinguished from mRNAs? It is to avoid this difficulty that we referred to them as bifRNAs (Dinger et al., 2008, 2011; Francastel and Hube, 2011; Ulveling et al., 2011a,b).

## Can we estimate the number of bifunctional RNAs in human?

While annotation systems are becoming more and more accurate, the previously automated annotated transcripts are being curated and verified manually. For example, the main features of the RefSeq collection (https://www.ncbi.nlm.nih.gov/books/NBK21091/) indicate, among others, non-redundancy, data validation, distinct accession series, and ongoing curation by NCBI staff and collaborators. In this type of nomenclature, “NM_” corresponds to validated protein-coding transcripts, while the prefix “NR_” indicates non-protein-coding transcripts also validated and curated. Each transcript, and more specifically each isoform produced, is associated with a unique identifier, which is itself linked to a gene locus. For example, the homo sapiens SRA1 gene has four isoforms that are deposited and accessible in NCBI website, variants 1 and 2 that are coding mRNAs and referred to as NM_001253764.1 and NM_001035235.3, respectively, whereas variants 3 and 4 are non-coding RNAs and identified as NR_045586.1 and NR_045587.1, respectively (Emberley et al., 2003; Chooniedass-Kothari et al., 2004, 2006). Actually, the diversity of SRA RNA transcripts in human cells is even more complex than described in PubMed since we have identified eight experimentally validated isoforms and at least 20 other isoforms from databases, all mainly non-coding transcripts (Hube et al., 2011), although they were not curated and thus, do not have an NR_ status.

We decided to use this characteristic (NM_ and NR_ criteria) to predict genes that could produce both coding and non-coding RNA isoforms. The methodology is explained in the legend of Figure 1. As seen in Figure 1B, 2,586 out of 28,048 gene loci are able to produce both NM_ and NR_ isoforms, i.e., almost 10% of the gene loci are able to produce both coding and non-coding isoforms that were validated and curated by NCBI. Going further in the analysis, we associated these transcripts to the “Affymetrix U133 plus 2 probes” and, as shown in Figure 1C, the majority of probes (66.7%) are unable to distinguish between coding and non-coding isoforms. It means that using the most up-to-date microarray technology, users are not able to distinguish between coding and non-coding genes for about 1,700 of them. Even if it represents only a small fraction of genes represented on the array (6%), data obtained from the array have to be used with caution, especially since the majority of these genes are genes expressed in almost all tissues [Jensen Tissues (Santos et al., 2015); Figure 1E] and are largely involved in metabolic pathways (KEGG hsa01100, z = −3.385, *p* = 0.0003563; not shown), which may lead to substantial biased interpretations. In addition, expression levels of these isoforms are not very different whether the transcripts are coding or not (Figure 1D) using dataset GDS4296 (Barrett et al., 2005).

This result leads to at least three very important conclusions. The first is that the non-coding versions of bifRNAs are not subjected to Nonsense-Mediated RNA Decay (NMD). Indeed, since these transcripts are detected in some cells/tissues, they escape the RNA surveillance mechanisms that otherwise rapidly degrades RNA with premature stop-codons (Zhang et al., 2009; Smith and Baker, 2015); Second, in contrast to what has been described for “conventional” ncRNAs supposed to date to be expressed at much lower levels than mRNAs (Ching et al., 2014; Wang et al., 2016), expression levels of coding vs. ncRNAs from the same locus are comparable regardless of their coding capacity (Figure 1D). Finally, almost two-third of the probes on expression arrays (Figure 1C) are not able to distinguish between coding and non-coding isoforms, which may lead to over- or mis-interpretation of data in some instances.

How to overcome this bias? An obvious but costly solution would be to develop new microarrays, taking into account all the annotated coding and non-coding isoforms. However, the constant discovery of new isoforms would rapidly overwhelm the process of probe design. The second solution may possibly come from the new technologies that are available today, such as high-throughput sequencing. However, at least in terms of isoform identification, this remains a challenge; the use of classical RNA-seq is still far from being accurate and the assembly of transcripts, although it is constantly being improved over time, still does not allow for true identification of the transcripts isoforms either. The latest generation of RNA-seq, the ultra-long reads method, seems to overcome most of these complications (Jain et al., 2017; Rhoads and Au, 2015), even if it remains to be improved since it is limited to several consecutive kbs, reveals error rates of about 10% and remains limited in the number of reads (Rhoads and Au, 2015).

## Conclusion

Since the burst of studies on non-coding RNAs, i.e., for almost 20 years now, there is no longer any doubt about their existence and importance in cellular processes. Now that the scientific community has accepted the concept of coding and non-coding RNAs, we may have to take a step backward to reassess the possibility that these two categories of transcripts are more interdependent than thought. These new conclusions impose for a deeper examination into the functional significance of these dynamic bifRNAs and for increased efforts toward a more integrated view of transcriptome/proteome in a given cellular context.

## Author contributions

All authors listed have made a substantial, direct and intellectual contribution to the work, and approved it for publication.

## Conflict of interest statement

The authors declare that the research was conducted in the absence of any commercial or financial relationships that could be construed as a potential conflict of interest.
